# The Comparison of Dental Anxiety between Patients Treated with Impacted Third Molar Surgery and Conventional Dental Extraction

**DOI:** 10.1155/2021/7492852

**Published:** 2021-09-04

**Authors:** Omur Dereci, Nesrin Saruhan, Gorkem Tekin

**Affiliations:** Department of Oral and Maxillofacial Surgery, Eskisehir Osmangazi University Faculty of Dentistry, Eskisehir, 26480, Turkey

## Abstract

**Background:**

The aim of the present study is to compare the dental anxiety levels between two outpatient clinics.

**Methods:**

Two hundred and seventy patients treated in two different clinics of minor oral surgery and dental extraction polyclinic in the Dental Faculty of Eskisehir Osmangazi University were included in the study. The impacted third molar surgery group and conventional dental extraction group consisted of 101 and 169 patients, respectively. The Modified Dental Anxiety Scale (MDAS) and Dental Fear Scale (DFS) were used to measure anxiety levels in patients treated in both clinics. Tests were made in an isolated room preoperatively. The differences in anxiety levels according to education status and gender were also evaluated.

**Results:**

The impacted third molar surgery group showed a significant increase in dental anxiety measured with DFS questionnaire (*p* < 0.05). However, MDAS revealed that there was no difference between anxiety levels between the impacted third molar surgery and conventional dental extraction groups (*p* > 0.05). There was also no difference in anxiety levels between patients with different education status (*p* > 0.05). Female patients demonstrated higher levels of anxiety in both MDAS and DFS indexes (*p* < 0.05).

**Conclusion:**

Dental anxiety may be higher in patients treated with impacted third molar surgery compared with conventional dental extraction. The education status of patients may not affect dental anxiety. Female patients may show increased levels of dental anxiety in conventional dental and impacted third molar extractions.

## 1. Introduction

Dental anxiety is a common concept that is defined as a mixture of feelings of unease, worry, and fear when a person encounters with dental treatment [1]. The use of surgical motors and handpieces and application of dental anesthesia mostly provoke fear and anxiety and cause discomfort in patients referred to a dental clinic. Dental anxiety may be a result of previous dental experiences or occurs in relation to previous traumatic events and a general state of anxiety independent from dental procedures [2, 3].

Surgical third molar extraction is a relatively advanced procedure compared to conventional tooth extraction and requires additional surgical instruments and postoperative set-up. Patients generally have an unpleasant notion as of the third molar removal surgery before they admit to the oral surgery clinic and expect a longer and complicated surgery when compared to conventional dental extraction. The psychological impact of third molar removal makes the operation stressful even if it is not a life-threating or major surgical procedure [4].

The null hypothesis of the current study proposes that there is no difference in the dental anxiety and fear perceived by the patients before third molar extraction and conventional dental extraction. The aim of the present study is to compare the dental anxiety levels treated with third molar removal and conventional dental extraction. The study is also aimed at comparing the dental anxiety between patients with different education status.

## 2. Materials and Methods

### 2.1. Patients

The study was approved by the local Clinical Research Ethics Committee with approval number 2020-469-12 and performed in accordance with the ethical standards laid down in the 1964 Declaration of Helsinki and its later amendments. Patients treated by dental extractions at the polyclinic and minor oral surgery clinic of Eskişehir Osmangazi University, Faculty of Dentistry, between 01 January 2018 and 01 June 2020 were included in the study. The patients treated with surgical third molar extraction were defined as the third molar group, and patients treated with conventional dental extraction were defined as the dental extraction group. The inclusion criteria of the study were as follows:

1. Patients older than 18
2. Patients with no noncontrolled systemic disease
3. Patients who were able to complete the questionnaire by themselves
4. Patients with impacted third molars that required bone removal (Pell-Gregory Classes II, III-A, B, and C) for extraction

Exclusion criteria were established as follows:

1. Patients under antianxiety and antipsychotic drug treatment with a history of mental illness

### 2.2. Conventional and Impacted Third Molar Extractions

Impacted third molar extractions were performed in the minor surgery operation room in which only minor oral surgical operations were made in an outpatient setting under sterile conditions. All cases with impacted third molars needed bone removal. Bone was removed around the impacted third molar with burs under copious physiological saline irrigation after a full-thickness envelope flap was reflected under local anesthesia. Tooth was divided into sections to ease the extract where needed. Antibiotics (1000 mg amoxicillin + clavulanic acid, 2 × 1), nonsteroidal anti-inflammatory drugs (naproxen sodium 550 mg, 3 × 1), and organ rinse (chlorhexidine digluconate 0.12%, 3 × 1) were prescribed for postoperative prophylaxis.

Conventional dental extractions were made under local anesthesia in the dental extraction polyclinic, in which all dental extractions that do not need any surgical intervention were performed. No postoperative prophylaxis was prescribed after nonsurgical dental extraction. Both dental and impacted third molar extractions were performed by one researcher (GT).

### 2.3. Anxiety Scoring

Dental Fear Scale (DFS) form was made of 20 separate statements that measure the fear level of the patient exposed to a dental procedure. Each statement was rated on a 5-point scale according to the patient's emotions and reaction to dental procedure agreement with that topic (1 point = not at all, 2 points = slightly so, 3 points = moderately so, 4 points = much so, and 5 points = very much so). Scores range between 5 (no fear) and 100 (intense fear).

Modified Dental Anxiety Scale (MDAS) questionnaire is an internationally accepted scale for dental anxiety [5] and consists of 5 multiple choice questions which include statements to measure dental anxiety level. The participant chooses the option which is closest to his/her feeling. Scores range between 5 (no anxiety) and 25 (maximum anxiety).

The validity and reliability of the Turkish version of MDAS [6] and DFS [7] have been demonstrated in previous studies. All patients were taken into an isolated room before the extraction and asked to fill both questionnaires by the same dental assistant. Besides the questionnaires, the informed consent forms were also signed by patients.

### 2.4. Statistical Analysis

An independent statistician reviewed the methodology and results of the study. SPSS version 22.0 Statistical Software (IBM, Chicago, USA) was used for statistical analysis of the results. Kolmogorov-Smirnov and Shapiro-Wilk tests (*p* < 0.05) showed that the measurement scores were not normally distributed. The significance of the difference in MDAS and DFS scores between the third molar and dental extraction groups was evaluated with the Mann-Whitney *U* test. The difference in MDAS and DFS scores between education status of the study patients was analyzed with the Kruskal-Wallis test, and the difference between genders was also evaluated with the Mann-Whitney *U* test. *p* < 0.05 was set as the significance level.

## 3. Results

The mean age was 30.12 ± 12.24. Ninety-five (35.2%) of the patients were male, and 175 patients were female. There were 2 cases of infection in the third molar group after the surgical operation. These cases were treated with antibiotherapy after surgical reentry to the third molar socket and debridement. There was one case of alveolitis in a patient who was a heavy smoker. This case was treated with curettage and saline irrigation of the alveolar socket.

DFS score was significantly increased in the third molar group compared to the dental extraction group (*p* < 0.05), whereas there was no significant difference between the 2 groups in terms of MDAS scoring (*p* > 0.05) (Table 1). The study patients were in a wide socioeconomic range. Eight of them had a preliminary school degree. Ninety of them graduated from high school, and 131 of them graduated from university. Eight of them had a postgraduate degree. There was no significant difference in MDAS and DFS scores between different education status (*p* > 0.05) (Table 2). Females showed a significant increase in MDAS and DFS scores in both the third molar and dental extraction groups (*p* < 0.05) (Table 3).

## 4. Discussion

Although several initiating and contributing factors of previous dental experience or unsubstantial comments of significant others on dental treatment were identified in the pathogenesis of dental anxiety, the exact mechanism and causality have not been established yet [5]. It is reported that patients who had previous experience with oral surgery had less anxiety compared to those who never received treatment with oral surgery [8]. Dental anxiety and fear are characterized by individual psychological and socioeconomic factors and the present oral health conditions of the patient [9]. The range and proportion of patients with diagnosed dental anxiety differ in various geographical regions. This difference may be a result of individual distinctions in the perception of dental treatment and oral surgery or diversity of the armamentarium used to measure and record the present condition of dental anxiety.

Conventional dental extraction is a fear-inducing dental treatment in the Turkish population. In a study with 160 participants with dental phobia, it is reported that dental extraction was the fourth most fear arousing among all dental applications [3]. A specific surgical armamentarium and surgical assistance are needed for impacted third molar extraction as different from conventional dental extraction. The increased number of surgical instruments and surgical preoperative set-up may be fearful and anxietic for the patient undergoing in impacted third molar surgery. de Jongh et al. [10] reported that third molar removal surgery had a minimal significant effect on the occurrence of dental anxiety or psychological trauma; however, they suggested that further studies are also needed to acquire data regarding dental anxiety levels after other types of surgical procedures. The rationale for the current study was to understand whether impacted third molar surgery evoked more increased dental anxiety compared to dental extraction, as contemplated in the routine outpatient application of dental extraction.

DFS is a well-known Dental Anxiety Scale and showed reliable results in displaying three elements of dental avoidance, physiological arousal, and fear of dental stimuli in dental anxiety [11]. Accordingly, the score obtained from DFS had an effect on short-term inflammation response after treatment [12]. Le et al. [12] used DFS to establish the anxiety levels of 59 patients before the impacted third molar removal and suggested that preoperative dental anxiety was correlated with short-term inflammatory responses of swelling and trismus after surgery. Similarly, it is reported that there is a significant correlation between dental anxiety and postoperative pain on the first day after impacted third molar surgery [5]. In the present study, DFS scores that were significantly increased in the third molar group may show that postoperative discomfort may be more prominent in impacted third molar surgery than conventional dental extraction. Interestingly, MDAS did not show a significant difference between the two groups, indicating that dental anxiety is at similar levels before impacted third molar surgery and conventional dental extraction. It is suggested that MDAS gives a general score for the dental anxiety of routine dental treatment, being not specific to oral surgical procedures [13]. In this context, the perception of the patient for both treatments may have been similar because both treatments are “tooth extractions” in nature. Patients may acknowledge the two treatments as equally anxietic in a common sense. At this point, the correlation of DFS and MDAS questionnaires before dental surgical procedures may be a topic of investigation in further studies.

Female gender has a predilection for dental anxiety as reported by many studies [2, 5, 14, 15]. In the current study, female patients showed significantly higher levels of dental anxiety compared to males. This condition is generally attributed to pain perception or pain threshold differences and differences in psychological aspects between the two genders. Relevantly, it is suggested that calm and relaxing communication between an oral surgeon and a female patient prior to the oral surgery was essential to reduce the degree of anxiety [5].

The education of the patient provides a prediction of his/her apprehension capacity and probable psychological response to the ongoing medical treatment [16]. Therefore, it is expected that patients with advanced education level show lower dental anxiety compared to patients with lower levels of education. Ragnarsson [17] conducted a population-based survey in an Icelandic population and suggested that patients with higher education have significantly lower levels of dental anxiety and fear during dental visits and have a lower incidence of total edentulousness. In the study of Eroglu et al. [7], STAI-trait, Dental Anxiety Scale, and Dental Fear Scale questionnaires were used to identify the dental anxiety levels in a sample of 200 patients from Turkey, and it was suggested that education and cultural level of the patient is more important than the economic ability in the development of dental anxiety and fear. However, the level of education did not show any difference for dental anxiety. The reason for that result may be the lower number of patients with postgraduate and preliminary education that is an important predictor as upper and lower bounds of the education level.

There are several limitations in the study. The first one may be the presence of two separate clinics in which the extractions were made. The minor oral surgery clinic was a separate clinic from all dental clinics in another building in our institution. This situation may have led to a feeling and worry that the treatment that is to be done in this isolated clinic is a major operation and more complicated than normal extraction for patients. This situation may have had an extra effect on the dental anxiety level of the patients in the third molar group. The second limitation is the evaluation of education status of patients without any record about the economic status. The establishment of the socioeconomic levels of the study patients would be a more appropriate approach for the comparison of dental anxiety.

## 5. Conclusion

DFS questionnaire showed that impacted third molar extraction may induce increased dental anxiety compared to conventional dental extraction. MDAS showed no significant difference between the two groups. The reason of this result may be that the MDAS is designed for general dental anxiety, not specific to oral surgery. Female patients may show increased dental anxiety compared to males. Education status of the patients may not affect dental anxiety before impacting third molars and conventional dental extraction.

## Figures and Tables

**Table 1:** DFA scores showed significant difference between the two groups. MDAS scores are not statistically significant between the two groups.

	MDAS (median ± std)	DFA (median ± std)
Third molar group (*n* = 101)	11 ± 4.58	40 ± 16.64
Dental extraction group (*n* = 169)	13 ± 4.16	40 ± 16.05
*p*	0.241	0.14

MDAS: Modified Dental Anxiety Scale; DFS: Dental Fear Scale.

**Table 2:** There were no statistically significant differences between different education status of study patients for DFS and MDAS scores.

	MDAS (median ± std)	DFS (mean ± std)
Preliminary school (*n* = 41)	12 ± 5.10	37 ± 15.93
High school (*n* = 90)	11 ± 4.40	40 ± 16.75
Graduate (*n* = 131)	12 ± 4.32	43 ± 16.41
Postgraduate education (*n* = 8)	8 ± 3.85	30.5 ± 14.31
*p*	0.422	0.271

MDAS: Modified Dental Anxiety Scale; DFS: Dental Fear Scale.

**Table 3:** Female patients showed increased dental anxiety compared to male patients for both MDAS and DFS scores.

	MDAS (median ± std)	DFS (median ± std)
Female	13 ± 4.46	44 ± 16.73
Male	10 ± 4.11	35 ± 14.76
*p*	<0.001	<0.001

MDAS: Modified Dental Anxiety Scale; DFS: Dental Fear Scale.

## Data Availability

The data used to support the findings of this study are available from the corresponding author upon request.

## References

[1] Buldur B., Armfield J. M. (2018). Development of the Turkish version of the Index of Dental Anxiety and Fear (IDAF-4C+): dental anxiety and concomitant factors in pediatric dental patients. *Journal of Clinical Pediatric Dentistry*.

[2] Lago-Méndez L., Diniz-Freitas M., Senra-Rivera C., Seoane-Pesqueira G., Gándara-Rey J. M., Garcia-Garcia A. (2006). Dental anxiety before removal of a third molar and association with general trait anxiety. *Journal of Oral and Maxillofacial Surgery*.

[3] Berggren U., Meynert G. (1984). Dental fear and avoidance: causes, symptoms, and consequences. *Journal of the American Dental Association*.

[4] López-Jornet P., Camacho-Alonso F., Sanchez-Siles M. (2014). Assessment of general pre and postoperative anxiety in patients undergoing tooth extraction: a prospective study. *The British journal of oral & maxillofacial surgery*.

[5] Xu J. L., Xia R. (2020). Influence factors of dental anxiety in patients with impacted third molar extractions and its correlation with postoperative pain: a prospective study. *Medicina Oral Patología Oral y Cirugia Bucal*.

[6] Tunc E. P., Firat D., Onur O. D., Sar V. (2005). Reliability and validity of the Modified Dental Anxiety Scale (MDAS) in a Turkish population. *Community Dentistry and Oral Epidemiology*.

[7] Eroglu C. N., Ataoğlu H., Küçük K. (2017). Factors affecting anxiety-fear of surgical procedures in dentistry. *Nigerian Journal of Clinical Practice*.

[8] Wong M., Lytle W. R. (1991). A comparison of anxiety levels associated with root canal therapy and oral surgery treatment. *Journal of Endodontics*.

[9] Armfield J. M., Pohjola V., Joukamaa M., Mattila A. K., Suominen A. L., Lahti S. M. (2011). Exploring the associations between somatization and dental fear and dental visiting. *European Journal of Oral Sciences*.

[10] de Jongh A., van Wijk A. J., Lindeboom J. A. (2011). Psychological impact of third molar surgery: a 1-month prospective study. *Journal of Oral and Maxillofacial Surgery*.

[11] Kleinknecht R. A., Thorndike R. M., McGlynn F. D., Harkavy J. (1984). Factor analysis of the dental fear survey with cross-validation. *Journal of the American Dental Association*.

[12] le S. H., Tonami K., Umemori S. (2021). Relationship between preoperative dental anxiety and short-term inflammatory response following oral surgery. *Australian Dental Journal*.

[13] Muglali M., Komerik N. (2008). Factors related to patients' anxiety before and after oral surgery. *Journal of Oral and Maxillofacial Surgery*.

[14] Heft M. W., Meng X., Bradley M. M., Lang P. J. (2007). Gender differences in reported dental fear and fear of dental pain. *Community Dentistry and Oral Epidemiology*.

[15] Malvania E. A., Ajithkrishnan C. G. (2011). Prevalence and socio-demographic correlates of dental anxiety among a group of adult patients attending a dental institution in Vadodara city, Gujarat, India. *Indian Journal of Dental Research*.

[16] Alkatheri A. M., Albekairy A. M. (2013). Does the patients' educational level and previous counseling affect their medication knowledge?. *Annals of Thoracic Medicine*.

[17] Ragnarsson E. (1998). Dental fear and anxiety in an adult Icelandic population. *Acta Odontologica Scandinavica*.

